# Genome Sequence of Avian *Escherichia coli* Strain IHIT25637, an Extraintestinal Pathogenic *E. coli* Strain of ST131 Encoding Colistin Resistance Determinant MCR-1

**DOI:** 10.1128/genomeA.00863-16

**Published:** 2016-09-01

**Authors:** Christa Ewers, Stephan Göttig, Maria Bülte, Sophie Fiedler, Manuela Tietgen, Ursula Leidner, Carsten Heydel, Rolf Bauerfeind, Torsten Semmler

**Affiliations:** a Institute of Hygiene and Infectious Diseases of Animals, Justus Liebig University, Giessen, Germany; b Institute of Medical Microbiology and Infection Control, Hospital of the Johann Wolfgang von Goethe-University, Frankfurt am Main, Germany; c Robert Koch Institute, Berlin, Germany

## Abstract

Sequence type 131 (ST131) is one of the predominant *Escherichia coli* lineages among extraintestinal pathogenic *E. coli* (ExPEC) that causes a variety of diseases in humans and animals and frequently shows multidrug resistance. Here, we report the first genome sequence of an ST131-ExPEC strain from poultry carrying the plasmid-encoded colistin resistance gene *mcr-1*.

## GENOME ANNOUNCEMENT

Extraintestinal pathogenic *Escherichia coli* (ExPEC) strains are a versatile group of bacteria revealing a complex phylogeny and considerable genomic plasticity. ExPEC strains cause a variety of disease syndromes, ranging from uncomplicated urinary tract infections to life-threatening bloodstream infections in humans and septicemia, peritonitis, and yolk sac infections in poultry (1, 2). Certain subpopulations of avian ExPEC have been suggested to be zoonotic agents (3, 4), which is of great concern, because they also have been associated with multidrug resistant clonal lineages, such as sequence type 131 (ST131) (2, 5).

We report the genome sequence of colistin-resistant avian ExPEC strain IHIT25637 carrying the plasmid-mediated *mcr-1* gene, which was described first in human and food animal samples in China in 2015 (6). IHIT25637 was isolated in Germany in October 2010 from the liver of a broiler chicken that had died of septicemia. Whole-genome sequencing was performed on a MiSeq sequencer using a MiSeq reagent kit v3 (Illumina, USA) resulting in 300 bp paired-end reads and an average coverage of 100×. Filtered 1.75 M clean reads were *de novo* assembled after quality trimming using SPAdes v3.7.1 (7) into scaffolds, and corresponding 90-fold coverage of the genome was generated. The draft genome sequence of *E. coli* IHIT25637 was 5,692,160 bp in size and had a G+C content of 50.4% in 183 contigs (maximum length, 402,413 bp; minimum length, 200 bp; *N*_50_ contig size 234,455 bp; mean coverage 90). The genome was annotated using the Rapid Annotation using Subsystem Technology (RAST) server (8). Annotation of this assembly identified 5,614 coding sequences (CDSs), 85 tRNAs, and 19 rRNAs.

The sequence type of IHIT25637 was identified as ST131 (https://cge.cbs.dtu.dk/services/), and its serotype was O25:H4 as determined by slide agglutination testing. Several virulence-related genes known to be associated with the ExPEC pathotype, like adhesin genes *csgA*, *fim*, *iha*, *tsh*, and *focA*, iron-acquisition related gene *chuA*, *iroN*, *eitA*, *etsB/C*, *fyuA*, *irp2*, *iucD*, and *iutA*, and invasion, serum resistance and capsule synthesis genes *ibeA*, *iss*, *kpsMTII*, and *neuC*, were present in strain IHIT25637. The allele type of the type I fimbrial adhesin gene was determined as *fimH*22, which has been associated with a fluoroquinolone-susceptible ST131-subgroup (9). Genome data revealed the presence of colistin resistance gene *mcr-1* which most likely conferred phenotypic resistance to the “last resort” antibiotics colistin and polymyxin B (MIC: 8 mg/L). *Mcr-1* was located on an IncHI2 plasmid (>200 kbp) of plasmid sequence type ST4 (10), which are major carriers of *mcr-1* in *E. coli* isolates from livestock and food and also from humans (11–13). In addition to *mcr-1*, the genome of IHIT25637 carried several resistance genes, such as *aph(3′)-Ic*, *aac(3)-IIa*, *aadA1*, *aadA2*, *strA* and *strB*, *bla*_TEM-1_, *cmlA1*, *catA1 sul1/2/3, dfrA1*, and *tet(A)*, but lacked genes for known extended-spectrum β-lactamases and carbapenemases. Only two ST131 *E. coli* strains carrying *mcr-1* have yet been described, namely, from chicken meat of European origin imported to Denmark in 2012 and from the urine of a human patient in Taiwan in 2014 (14, 15). However, the appearance of *mcr-1*-harboring, colistin resistant ExPEC belonging to the epidemiological successful clonal lineage ST131 poses a serious public health threat.

### Accession number(s).

This whole-genome shotgun project has been deposited at DDBJ/EMBL/GenBank under the accession number MAIV00000000. The version described in this paper is version MAIV00000000.1.
